# Shisha microbiota: the good, the bad and the not so ugly

**DOI:** 10.1186/s13104-018-3553-9

**Published:** 2018-07-06

**Authors:** Julia Hani, Ghenwa Abdel Nour, Joanne Matta, Boushra Jazzar, Michael W. Pfaffl, Lara Hanna-Wakim, Afif M. Abdel Nour

**Affiliations:** 1 Faculty of Agricultural and Food Sciences, The Holy Spirit University of Kaslik, Jounieh, Lebanon; 2 Institute of Animal Physiology & Immunology, Faculty of Life Sciences, Technical University of Munich, Freising, Munich, Germany

**Keywords:** Microbiota, Bacteria, Shisha, Tobacco, Public health

## Abstract

**Objective:**

Over the last decade, there has been a rapid expansion of the trendy water pipe smoking around the world especially among younger adults. The initial objective of this study was to identify the microbiota of the shisha, which may either be of no harm for the smoker or enhance the threat on his well-being. The total DNA for the metagenomics study was conducted on three different shishas from three different delivery shops in Jounieh, Lebanon. The microbiota in two solid parts of the shisha, shaft and hose, were analysed including the fresh tobacco and the water in the bowl. All samples were analysed using high-throughput sequencing of 16S rRNA gene amplicons.

**Results:**

Overall, more than 40 bacterial genera were found in the three investigated shishas, some are commensal others are pathogenic. All three shishas showed similar microbial content regarding the bacteria inhabiting in water, shaft, or hose. From the results of this study it appears that a very large quantity of bacteria was found in the water pipes, some are harmful and others beneficial. We assume that the presence of gut dependent microbiota is related to the loose hygienic conditions in which the shisha is prepared.

## Introduction

Water pipe smoking, known as shisha, hookah, nargileh, argileh or “Hubbly-bubbly” depending on the country, is a trendy form of tobacco smoking spreading widely around the world, which has gained enormous popularity, especially in the Middle-East among college and university students [1–3].

Shisha has recently grown to become desired in low-income as well as high-income countries, despite the well-known impact of tobacco on the smoker’s health [4].

Water pipe smoking is a process that uses a specific device with different components: the head, the body and the bowl. The head contains tobacco, covered with aluminium foil where coal is placed. The body consists of a shaft that connects the head to the bowl filled with water. A hose is attached to the bowl connecting the smoker to the whole system [5].

According to the World Health Organization (WHO) (2005), in the bottom of the head of the water pipe there are small holes through which smoke passes into the body’s central gasket. The smoke that is puffed by the smoker is passed down from the tube and then through the jar of water before it is inhaled by the smoker.

A common misconception that might be lurking around is that the water filters the inhaled air, which makes consumers believe it’s healthier than cigarette smoking [6]. In fact, even with the same concentration of nicotine in cigarettes and shisha, a shisha smoker is subject to higher threats because of the duration of exposure to nicotine and other chemicals: a single shisha session could last up to 2 h [5, 7].

Shisha smoking is also associated with cardiovascular diseases, hyperglycaemia as well as low antioxidant capacity and vitamin C level [8].

The purpose of this study is to identify the microbiota in every part of the shisha. To our knowledge this is the first time this analysis is done.

## Main text

### Methods

Total DNA for the metagenomic study was extracted from three different shishas. The later were delivered three different shisha delivery shops. We analyzed the microbiota in four parts of the shisha was done as follows:

1. The fresh tobacco (before heating it with charcoal) is put in the head. The tobacco, the shaft, the hose and the tip were rinsed with ultrapure sterile water and were subjected for a DNA extraction.
2. The water in the bowl was directly collected in 50 ml tubes for DNA extraction.

High-throughput sequencing of 16S rRNA gene amplicons.

Water samples, collected from the 4 different parts described above, were thawed on ice, suspended in 600 ml DNA stabilization buffer (Stratec Biomedical) and in 400 ml phenol/chloroform/isoamyl alcohol (25:24:1 v/v Sigma-Aldrich). The containing cells were mechanically lysed on ice with 500 mg glass beads (0.1 mm Roth) by a bead-beater (MP Biomedicals).

After a subsequent heat treatment (8 min @ 95 °C) and centrifugation (16,000 g; 5 min @ 4 °C), 150 ml supernatant was incubated (20 min @ 37 °C) with 15 ml ribonuclease (0.1 mg/ml; Amresco), and finally centrifuged (550 g; 30 min @ room temperature). ZymoBIOMICS™ DNA Mini Kit (Zymo Research, USA) was used to extract DNA according to the manufacturer instructions. DNA concentration (260 nm) and purity (260 nm/280 nm ratio) were evaluated in 1 µl solution with the NanoDrop 1000 system (Thermo Fisher Scientific). If not processed immediately, samples were stored frozen at − 20 °C.

The preparation of amplicon libraries of was done following the method the 16S rRNA V3–V4 region and sequencing was performed as described in detail previously [9]. Data were analysed as described previously [9]. Raw reads were processed with the “Integrated Microbial Next Generation Sequencing” (IMNGS) pipeline (http://www.imngs.org) [10] based on the UPARSE approach [11].

### Results

**Fig. 1:**
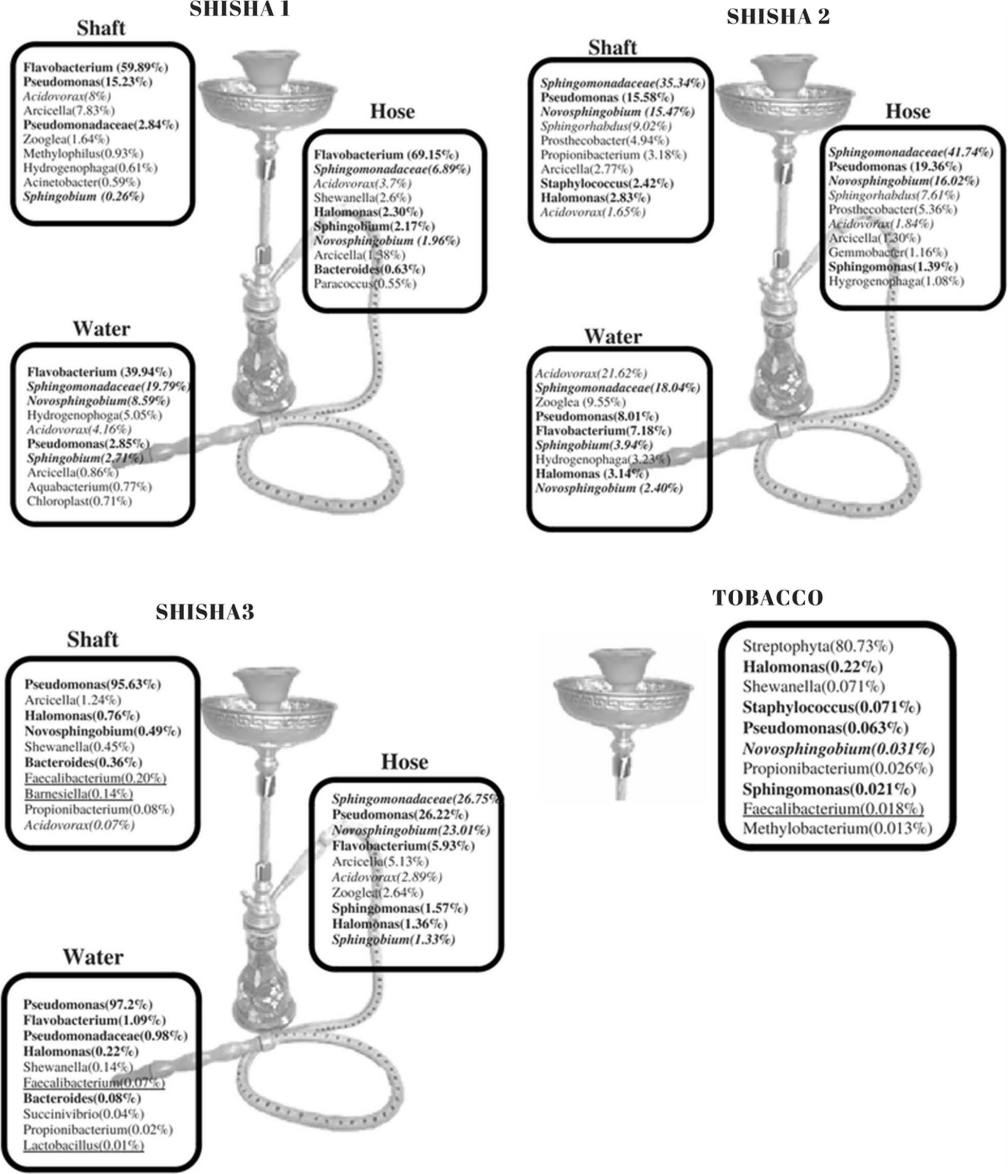
Microbial results of the tests on the shishas and on the tobacco, pathogenic bacteria are depicted in bold and gut microbiota are underlined

**Fig. 2:**
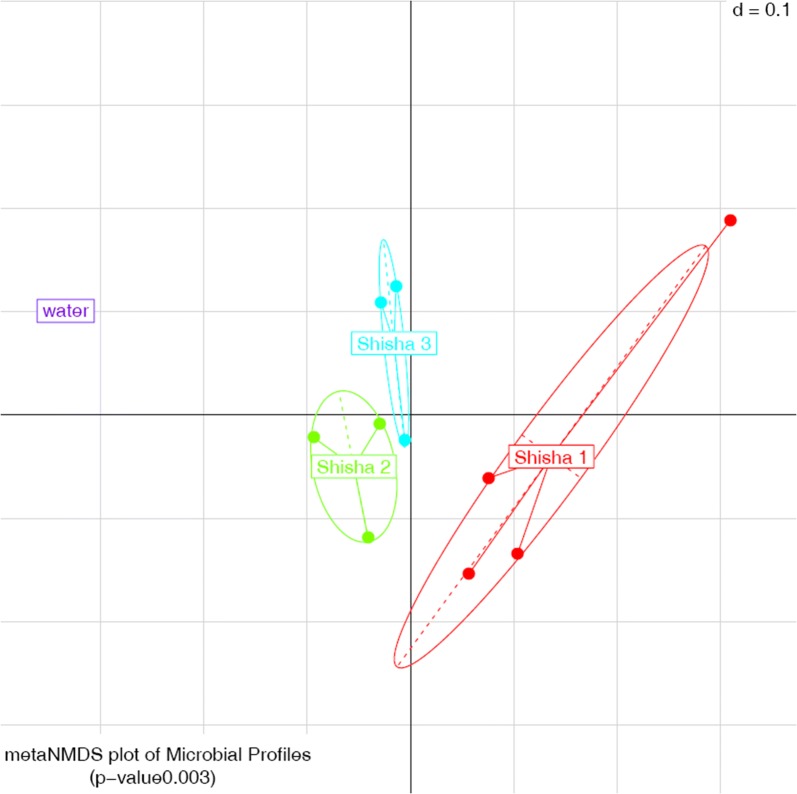
Microbiota clustering of the three shisha samples and the control water

Results found more than 40 genera on the 3 shishas, among those were commensal and pathogenic bacteria (Fig. 1). The percentage of each bacterium found is calculated relatively to its location on the shishas studied. The rates shown below show the highest amount of one genus at a specific location in one of the three shishas. The part of the three shishas studied showed a strong clustering (Fig. 2).

Bacteria found in shishas can be grouped into three categories: pathogenic, aromatic compound degradators and others, among which gut bacteria were found. Out of all beneficial bacteria encountered, most abundant were *Novosphingobium* (23.01% in shisha 3), *Acidovorax* (21.62% in shisha 2), followed by Sphingomonadaceae (41.74% in shisha 2), *Sphingobium* (2.71% in shisha 1), *Novosphingobium* (2.40% in shisha 2).

On the other hand, among the pathogenic bacteria, *Flavobacterium* was the most abundant (69% in shisha 1), *Halomonas* (2.3% in shisha 1), followed by *Staphylococcus* (2.42% in shisha 2), then *Pseudomonas* (97.2% in shisha 2), *Bacteroides* (0.36% in shisha 3).

Bacteria from the human gut microbiota were found in shisha 3, such as *Faecalibacterium* (0.20%) and *Lactobacillus* (0.01%), *Barnesiella* (0.14%). *Faecalibacterium* was found in tobacco as well (0.018%).

Overall, all three shishas showed similar content regarding the bacteria inhabiting the water, the shaft and the hose, but each one had a dominance of a specific genera.

Shisha 1 has a predominance of *Flavobacterium* with 39.94% in the water, 69.15% in the hose and 59.89% in the shaft. On the other hand, shisha 2 shows high contamination with Sphingomonadaceae at the rate of 18.04% in the water, 41.74% in the hose and 35.34% in the shaft. As for shisha 3, the most abundant are *Pseudomonas* at 97.20% in the water 26.22% in the hose and 95.63%. in the shaft.

Concerning the tobacco, Streptophyta invades the whole compartment at a rate of 97%, with traces of *Staphylococcus* and *Pseudomonas* along with other bacterial minorities.

### Discussion

It is well known that tobacco smoking in all its forms has negative effects on the human health and wellbeing, but shisha comes with different threats: water pipe smoking is a pattern of smoking tobacco using a specific device which provides some adequate conditions in terms of humidity, temperature and light exposure to allow bacterial proliferation, thus exposing the smoker to those bacteria. In addition, having different physical states allows a larger spectrum of bacteria to live and multiply as proven by the results. Among the bacteria found, some of them belonged to environmental microbiota, others to soil and water, and human microbiota as well.

Some of these are part of the natural microbial flora of water and environmental air [12]. Natural water microbiota includes *Pseudomonas* and *Flavobacterium* which explains the high occurrence of these in most of the shishas. But on the other hand, microbial flora in the air is not constant [12]: air does not provide suitable conditions for bacterial growth, thus bacteria can be introduced by any source of contamination and then transmitted to exposed individuals causing various infections in the respiratory tract.

Sphingomonadaceae is a family of 11 genera, among those are *Sphingobium*, *Sphingomonas* and *Novosphingobium* found in all 3 shishas at different rates [13]. These genera are found in abundance in soil or water, and are considered human pathogens, able to induce infections on healthy smokers as well as immunocompromised individuals [13]. *Pseudomonas* is an organism encountered in the environment in soil and water, and was found to be elevated in shisha 2. This bacterium is only a threat for immunocompromised individuals with milder consequences on healthy smokers [14].

*Propionibacterium*, *Staphylococcus* and *Acinetobacter* are part of the skin microbiota. In specific environmental conditions, these are responsible for skin disease, of which, acne [15]. The intestine and urinary system contain *Bacteroides*, *Lactobacillus*, *Staphylococcus*, and *Pseudomonas*. All these commensal bacteria are capable of causing infections when their environment changes, therefore their presence on the water pipes is considered a threat to the smoker’s health. *Pseudomonas* invades wounds and burns on a dermal level, and causes septicaemia and lung infections by invading the respiratory system [16]. *Staphylococcus* is a bacterium of the human dermal flora, and is found as well in water [17]. *Staphylococcus* can cause health threats by multiplication in the tissues where it produces toxins and enzymes. Such events may lead to pneumonia as well as several other diseases [15]. Less abundant bacteria, like *Clostridium* and *Bacteroides* are also responsible of some infections and diseases because of their pathogenicity [12]. Along with these pathogens, some others contribute to enhance the shisha’s environment.

Tobacco smoke produces several kinds of xenobiotics released in the air [18]. By definition; xenobiotics are foreign chemicals unrecognizable by the human body, which become toxic at high concentrations. Many of the bacteria found in the shishas, such as *Novosphingobium*, *Sphingomonas*, *Acidovorax* and *Methylobacterium* are considered biodegradators of xenobiotics improving the quality of the air to which the smoker is exposed to [13, 19].

*Methylobacterium* can disrupt low molecular weight hydrocarbons including isoprene found in the smoke resulting from shisha. Isoprene is a carcinogenic molecule that can create dermal damage as well as respiratory diseases [20].

*Novosphingobium* and *Sphingomonas* present opposite aspects, although pathogenic bacteria, they have a role in biodegradation of environmental contaminants.

### Conclusion

Our results showed that a large spectrum of bacteria could be identified on any device used in water pipe smoking. Some were found to be part of the harmful bacteria whereas others were found to be related to the beneficial ones where some are harmful and others beneficial. The difference in the rate depends on the hygienic conditions in which the shisha is prepared limiting the contamination rate, and reducing health threats that are added to the original threats of smoking tobacco. The presence of human gut or soil microbes inside the shisha alerted us on the fact that hygienic procedures similar to food preparation should be applied to shisha preparations. This includes washing hands regularly and more frequently. It is recommended to use a plastic hose, to perform a regular intensive cleaning using hot water, and time to time full decontamination could limit the spreading of such microbes.

## Limitations

We acknowledge that the small number of shishas is a limitation for our study. But this is a pilot study that will help future scientist study the microbiota environment in shisha. We acknowledge that our study is shedding the light on a dark yet very interesting point towards the understanding of the health impact of the shisha smoking. Many bacteria that we found are still unknown and future studies will help understanding the responsibility of microbiota in shisha.

## Abbreviations

WHO: World Health Organization
DNA: deoxyribonucleic acid
rRNA: ribosomal ribonucleic acid

## References

[1] Daou KN, Bou-Orm IR, Adib SM (2017). Factors associated with waterpipe tobacco smoking among Lebanese women. Women Health.

[2] Mahboub B, Mohammad AB, Nahle A, Vats M, Al Assaf O, Al-Zarooni H (2018). Analytical determination of nicotine and tar levels in various dokha and shisha tobacco products. J Anal Toxicol.

[3] Maziak W, Taleb ZB, Bahelah R, Islam F, Jaber R, Auf R, Salloum RG (2015). The global epidemiology of waterpipe smoking. Tob Control.

[4] Aslam HM, Saleem S, German S, Qureshi WA (2014). Harmful effects of shisha: literature review. Int Arch Med.

[5] Awan KH, Siddiqi K, Patil S, Hussain QA (2017). Assessing the effect of waterpipe smoking on cancer outcome—a systematic review of current evidence. Asian Pac J Cancer Prev.

[6] Schubert J, Kappenstein O, Luch A, Schulz TG (2011). Analysis of primary aromatic amines in the mainstream waterpipe smoke using liquid chromatography–electrospray ionization tandem mass spectrometry. J Chromatogr A.

[7] Kadhum M, Sweidan A, Jaffery AE, Al-Saadi A, Madden B (2015). A review of the health effects of smoking shisha. Clin Med.

[8] Wong LP, Alias H, Aghamohammadi N, Aghazadeh S, Hoe VC (2016). Shisha smoking practices, use reasons, attitudes, health effects and intentions to quit among shisha smokers in Malaysia. Int J Environ Res Public Health.

[9] Lagkouvardos I, Klaring K, Heinzmann SS, Platz S, Scholz B, Engel KH, Schmitt-Kopplin P, Haller D, Rohn S, Skurk T (2015). Gut metabolites and bacterial community networks during a pilot intervention study with flaxseeds in healthy adult men. Mol Nutr Food Res.

[10] Lagkouvardos I, Joseph D, Kapfhammer M, Giritli S, Horn M, Haller D, Clavel T (2016). IMNGS: a comprehensive open resource of processed 16S rRNA microbial profiles for ecology and diversity studies. Sci Rep.

[11] Edgar RC (2013). UPARSE: highly accurate OTU sequences from microbial amplicon reads. Nat Methods.

[12] Cabral JP (2010). Water microbiology. Bacterial pathogens and water. Int J Environ Res Public Health.

[13] Lin JN, Lai CH, Chen YH, Lin HL, Huang CK, Chen WF, Wang JL, Chung HC, Liang SH, Lin HH (2010). *Sphingomonas paucimobilis* bacteremia in humans: 16 case reports and a literature review. J Microbiol Immunol Infect.

[14] Hardalo C, Edberg SC (1997). *Pseudomonas aeruginosa*: assessment of risk from drinking water. Crit Rev Microbiol.

[15] LeChevallier MW, Seidler RJ (1980). *Staphylococcus aureus* in rural drinking water. Appl Environ Microbiol.

[16] Van Nevel S, Koetzsch S, Proctor CR, Besmer MD, Prest EI, Vrouwenvelder JS, Knezev A, Boon N, Hammes F (2017). Flow cytometric bacterial cell counts challenge conventional heterotrophic plate counts for routine microbiological drinking water monitoring. Water Res.

[17] Antai SP (1987). Incidence of *Staphylococcus aureus*, coliforms and antibiotic-resistant strains of *Escherichia coli* in rural water supplies in Port Harcourt. J Appl Bacteriol.

[18] Adams JD, O’Mara-Adams KJ, Hoffmann D (1987). Toxic and carcinogenic agents in undiluted mainstream smoke and sidestream smoke of different types of cigarettes. Carcinogenesis.

[19] Ochsner AM, Sonntag F, Buchhaupt M, Schrader J, Vorholt JA (2015). *Methylobacterium extorquens*: methylotrophy and biotechnological applications. Appl Microbiol Biotechnol.

[20] Srivastva N, Vishwakarma P, Bhardwaj Y, Singh A, Manjunath K, Dubey SK (2017). Kinetic and molecular analyses reveal isoprene degradation potential of *Methylobacterium* sp. Bioresour Technol.

